# Association Between Cancer Screening Patterns and Carer Literacy in Individuals With Cognitive Decline: An Observational Study

**DOI:** 10.1002/cam4.70311

**Published:** 2024-10-23

**Authors:** Yujiro Kuroda, Aya Goto, Kazuaki Uchida, Taiki Sugimoto, Kosuke Fujita, Yoko Yokoyama, Takeshi Nakagawa, Tami Saito, Taiji Noguchi, Ayane Komatsu, Hidenori Arai, Takashi Sakurai

**Affiliations:** ^1^ Department of Prevention and Care Science Center for Development of Advanced Medicine for Dementia, Research Institute, National Center for Geriatrics and Gerontology Obu Japan; ^2^ Department of Global Health and Population Harvard T. H. Chan School of Public Health Boston Massachusetts USA; ^3^ Center for Integrated Sciences and Humanities Fukushima Medical University Fukushima Japan; ^4^ Department of Medicine University of Washington Seattle Washington USA; ^5^ Department of Human Sciences Osaka University Suita Japan; ^6^ Department of Social Science Center for Gerontology and Social Science, Research Institute, National Center for Geriatrics and Gerontology Obu Japan; ^7^ National Center for Geriatrics and Gerontology Obu Japan; ^8^ Department of Cognition and Behavior Science Nagoya University Graduate School of Medicine Nagoya Japan

**Keywords:** cancer screening, carer health literacy, decision‐making, health communication, health literacy, healthcare barriers, mild cognitive impairment, public health policy, screening guidelines

## Abstract

**Objective:**

The incidence rates of dementia, mild cognitive impairment, and cancer increase with age, posing challenges to affected individuals and their families. However, there are currently no clear cancer screening guidelines for individuals with cognitive impairment. This study analyzed the impact of carer health literacy on screening behaviors in this population.

**Methods:**

We conducted a postal follow‐up survey, associated with the National Center for Geriatrics and Gerontology—Life STORIES of People with Dementia, that targeted primary carers to assess their reports regarding patient attendance at regular cancer screenings recommended by the Japanese Ministry of Health, Labor and Welfare, over the preceding 2 years. Screening rates were compared between the memory clinic cohort and the national average, and the influence of carer health literacy level on screening was analyzed.

**Results:**

Among the 826 total individuals analyzed, the memory clinic cohort exhibited lower breast cancer screening rates, at 11% among female patients aged 65–74 years versus the national average of 32%. Higher health literacy among carers was significantly associated with increased screening. For female patients, carers with high levels of communicative health literacy were more likely to ensure that patients attended screenings for gastric (adjusted odds ratio [AOR], 1.77; 95% confidence interval [CI], 1.03–3.04), colorectal (AOR, 1.70, 95% CI 1.08–2.70), and breast cancers (AOR, 3.08; 95% CI, 1.40–6.76). Among the male patients, high communicative health literacy was associated with increased lung cancer screening attendance (AOR, 1.82; 95% CI, 1.11–2.99).

**Conclusions:**

Our research highlights a notable gap in cancer screening attendance between individuals with cognitive impairment and the general population, potentially arising from the intricate nature of screening procedures and the extensive burden on carers. More informed decisions and increased screening rates can be achieved through patient‐centric communication strategies that accommodate the cognitive abilities of patients, ensuring the comprehensibility and accessibility of health‐related information.

## 1 Background

The incidence rates of dementia, mild cognitive impairment (MCI), and cancer increase with age, presenting a complex challenge for individuals with cognitive impairment and their families [1]. In particular, as cognitive decline progresses, questions may arise regarding the value of continued cancer screening [2, 3]. Given the significant correlation between an individual's level of health literacy and that of their family members, we hypothesized that health literacy might serve as a social network resource that influences health‐related behaviors among individuals with cognitive impairment [4]. This association underscores the importance of addressing the psychological and practical aspects of cancer screening in this population, in order to balance the benefits of early detection with the nuanced needs of individuals with cognitive impairment and their carers.

**FIGURE 1:**
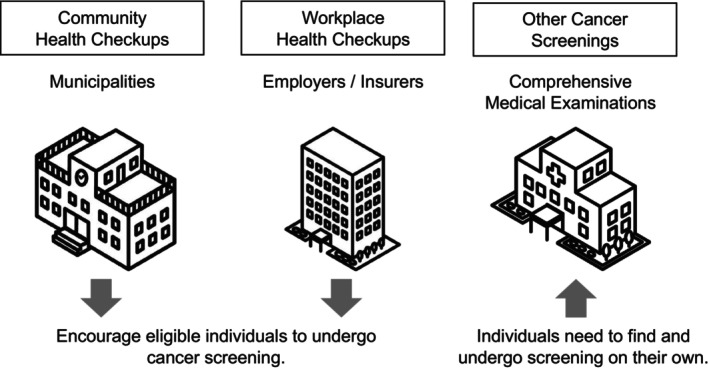
Overview of common cancer screening processes in japan (community, workplace, and other cancer screenings).

In Japan, the Ministry of Health, Labor, and Welfare (MHLW) recommends specific cancer screening schedules for individuals based on sex and age. Men are advised to undergo gastric, lung, and colorectal cancer screenings from the ages of 50, 40, and 40 years, respectively; while women are recommended to undergo cervical and breast cancer screenings from the ages of 20 and 40 years, respectively. Annual colorectal and lung cancer screenings are advised, whereas screenings for all other cancer types are recommended biennially [5]. Figure 1 provides an overview of the different settings where cancer screenings are typically conducted in Japan—including community health checkups, workplace health checkups, and other types of screenings. However, guidelines for individuals with dementia, particularly those with severe dementia, remain inconsistent and unclear [3]. For example, while some studies suggest limited benefits of such screening in persons with severe dementia [6], there is less consensus regarding persons with MCI or mild dementia. Individuals with dementia generally have a shorter life expectancy than that of the general population [7, 8]; however, recent data suggest that life expectancy post‐diagnosis may be increasing [9, 10], with individuals with MCI experiencing longer survival times. In our cohort of patients in a Japanese memory clinic, those with MCI showed a median survival rate of > 3000 days, indicating lower mortality rates than those of individuals with dementia [11]. While pneumonia is reportedly a common cause of death, our cohort had more deaths attributed to cancer [11], indicating the importance of cancer screening in patients of memory clinics.

As cognitive function declines, affected individuals rely on carers for decision‐making—particularly as their ability to perform daily activities decreases [12, 13]. Carers play a critical role in navigating complex healthcare decisions, including cancer screenings. This relationship substantiates our use of carers' health literacy as a proxy for patient screening behaviors in this study. Cognitive decline can make more complex breast cancer screenings, such as mammography, particularly challenging [1]. Carers often experience decisional conflict and increased burden, potentially owing to a lack of both knowledge and confidence regarding making decisions related to mammography for relatives with Alzheimer's disease and related dementias [14, 15, 16]. Ishikawa et al. indicated that health literacy within a family is interrelated, highlighting the significance of health literacy in carers [4].

Therefore, this study focused on health literacy in carers, which has been shown to be associated with better health‐related decision‐making and outcomes, as well as reduced burden of care [17, 18, 19, 20]. We hypothesized that health literacy in carers influences cancer screening behaviors in patients and that adequate information and support for carers might encourage better participation in cancer screening. This study specifically aimed to (1) compare cancer screening attendance rates between the general Japanese population and a patient cohort from a Japanese memory clinic cohort and (2) analyze the impact of carer health literacy on the screening behaviors of the individuals they care for.

## 2 Methods

### 2.1 Study Cohort

This study was a follow‐up survey to the Japanese National Center for Geriatrics and Gerontology (NCGG)—Life STORIES of Individuals with Dementia study [11]. This represents a comprehensive dataset that includes clinical records and prognostic data from patients diagnosed with various types of dementia or MCI who visited the NCGG's memory clinic between July 2010 and September 2018. In December 2022, we expanded the target period and number of participants to include patients who visited the clinic between July 2010 and July 2022—allowing us to incorporate more recent data concerning cancer screening behaviors within this population, focusing specifically on the influence of carer health literacy. Ethical approval for this study was obtained from the institutional review board (IRB) of the NCGG (approval number: 1661).

### 2.2 Participants

Participants were drawn from a cohort of 5148 patients aged ≥ 65 years who visited the NCGG's memory clinic between July 2010 and July 2022. Primary carers were surveyed to report on their patients' cancer‐screening behaviors. We excluded responses from the patients themselves, non‐primary carers, and individuals with unspecified relationships. We also excluded carers of patients who were deceased or had moderate‐to‐severe dementia, as the study focused only on patients with MCI or mild dementia, where cancer screening remains relevant. Responses with missing information concerning the outcome or main explanatory variables were also excluded, resulting in a final analytical cohort of 826 primary carers.

### 2.3 Measurements

#### 2.3.1 Outcome

The primary outcome was the cancer screening attendance rate among the patients over the preceding 2 years, as reported by the primary carers through their responses to a questionnaire. This approach ensured accurate data collection from the population with cognitive decline, who might not have accurately recalled their screening attendance rates. The questionnaire mirrored the screening items recommended by the Japanese MHLW, including gastric, lung, colorectal, cervical, and breast cancer screenings [5]. Prostate cancer, although prevalent among males in Japan, was not included in this study because it is not part of the MHLW‐recommended screenings for this target population. To ensure comparability with national data, these screenings were aligned with the Comprehensive Survey of Living Conditions [21] conducted by the MHLW, which excludes individuals with dementia. Each cancer screening type was defined using the following specific diagnostic methods: (1) barium X‐ray radiography or endoscopy (gastroscopy or fibroscopy) for gastric cancer; (2) chest X‐ray radiography and sputum tests for lung cancer; (3) fecal occult blood tests for colorectal cancer; (4) Pap smear for cervical cancer; and (5) mammography or breast ultrasonography for breast cancer. This approach facilitated accurate recall and reporting by carers, enhancing data reliability. Our subsequent comparison of the cohort's screening attendance rates with publicly available national data aimed to contextualize our findings within the broader landscape of cancer screening in Japan—while noting the exclusion of individuals with dementia from the national survey [21].

As part of our questionnaire, the primary carers were asked to indicate any reasons they had for not enforcing regular cancer screenings in their patients, by selecting from 14 possible options. These options were consistent with those used in the Comprehensive Survey of Living Conditions. They consisted of “Didn't know about it”; “No time”; “Location was too far”; “Costs money”; “Anxious about tests (bloodwork, endoscopy)”; “Was going to/admitted at a medical facility at the time”; “Don't feel need to get it annually”; “Confident in health, don't feel need”; “Can go to the doctor anytime if needed”; “Worried about results, don't want to get it”; “Too much hassle”; “Concerned about COVID‐19 infection”; “Missed opportunity due to COVID‐19 impact”; and “Other.” The carers were instructed to select all applicable reasons.

#### 2.3.2 Exposure

The carers' levels of communicative and critical health literacy (CCHL) were assessed using a questionnaire that focused on their levels of confidence regarding managing health‐related information. The CCHL scale is based on a validated questionnaire developed by Ishikawa et al. in 2008 [24] for the Japanese population [22]. It has been extensively validated and is widely used in public health research in Japan, thus indicating its external validity in this context [4, 23]. Communicative health literacy involves the ability to seek, understand, and use health‐related information to address health problems—including the skills to collect, comprehend, and communicate health‐related needs effectively [24]. Critical health literacy entails higher‐level skills for analyzing health information, evaluating source credibility, and making informed decisions based on critical assessments [25]. The scale consists of five items, with three measuring communicative health literacy and two measuring critical health literacy, rated on a 5‐point scale from 1 (never) to 5 (strongly agree). It has demonstrated high internal consistency (Cronbach's alpha = 0.86), with all item‐total correlations being positive (0.77–0.85). The CCHL scores of our cohort were dichotomized into low and high groups, based on the median scores. This decision to use median scores follows the methodology established by Ishikawa et al. and subsequent studies that used the CCHL scale [4]. The cutoffs of 11 for communicative health literacy (items i–iii) and 7 for critical health literacy (items iv and v) were determined based on these median values [22].

#### 2.3.3 Patient‐Related Variables

**FIGURE 2:**
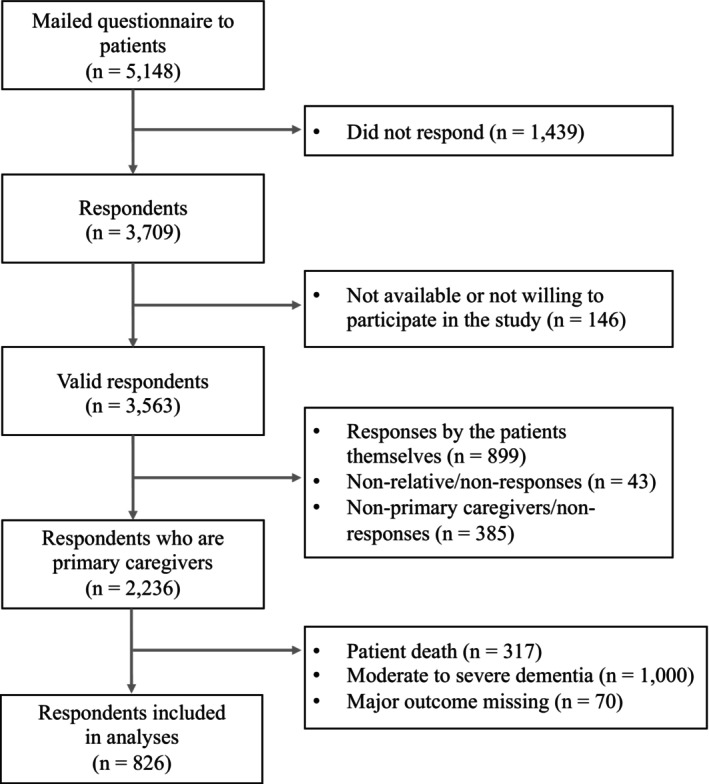
Selection of participants. Moderate‐to‐severe cognitive impairment was determined based on the carers' questionnaire responses, with “moderate” defined as needing assistance with personal care or having difficulty with dressing or eating independently and “severe” as requiring constant care and having significant communication difficulties. These individuals were excluded.

Information regarding the patients—such as age at the study's commencement, sex, and dementia type at initial diagnosis—was extracted from the NCGG memory center's database. Dementia types were determined by specialist physicians using neuropsychological assessments to classify cognitive functions. The MCI and dementia definitions were based on the criteria set by the US National Institute on Aging‐Alzheimer's Association. Dementia was categorized as Alzheimer's disease [26, 27], dementia with Lewy bodies or Parkinson's disease [28], vascular dementia [29], and frontotemporal lobar degeneration [30]. Besides initial diagnosis data, carers were asked to evaluate the current cognitive states of their patients, selecting from “suspected dementia (no daily life impairment but a feeling of cognitive dysfunction)”; “mild (occasional reminders or supervision needed, with some forgetfulness concerning recent events or upcoming tasks)”; “moderate (assistance needed with personal care, difficulty with dressing or eating independently)”; or “severe (constant care required, significant communication difficulties)”; to provide an up‐to‐date reflection of the patient's condition. This carer‐reported cognitive state was crucial for determining the inclusion and exclusion of participants, as is shown in Figure 2. The specialist‐determined dementia type was used primarily as a reference for initial diagnosis (Table 1).

**TABLE 1:** Basic characteristics of participants, distributed by sex.

	Total (*n* = 826)	Patient's sex		*p*
		Female (*n* = 517)	Male (*n* = 309)	
Patient‐related information				
Age (years), median (IQR)	83. 0 (79.0–87.0)	84.0 (80.0–88.0)	82.0 (77.0–86.0)	< 0.01
Age group (years), *n* (%)				
65–74	90 (10.9)	45 (8.7)	45 (14.6)	< 0.01
75–84	381 (46.1)	219 (42.4)	162 (52.4)	
≥ 85	355 (43.0)	253 (48.9)	102 (33.0)	
Medical information				
Long‐term Care Certification				
Independent	237 (28.7)	105 (20.3)	132 (42.7)	< 0.01
Support Required to Long‐Term Care	397 (48.1)	278 (53.8)	119 (38.5)	
Level 1	170 (20.6)	122 (23.6)	48 (15.50)	
Long‐term care Level 2 or higher				
Initial diagnosis				
Subjective cognitive impairment	72 (8.7)	47 (9.1)	25 (8.1)	< 0.01
Mild cognitive impairment	295 (35.7)	174 (33.7)	121 (39.2)	
Alzheimer's disease	286 (34.6)	200 (38.7)	86 (27.8)	
Other dementia types	54 (6.5)	26 (5.0)	28 (9.1)	
Non‐specified dementia	119 (14.4)	70 (13.5)	49 (15.9)	
Follow‐up period from initial diagnosis to survey, median (IQR)	1346.5 (728.0–2546.5)	1372.0 (769.0–2716.0)	1288.0 (670.5–2314.5)	0.037
Primary carer‐related information				
Age (years), median (IQR)	69.0 (59.0–80.0)	65.0 (57.0–79.0)	74.0 (62.5–80.0)	< 0.01
Age group (years), *n* (%)				
≤ 64	358 (43.3)	265 (51.3)	93 (30.1)	< 0.01
65–74	165 (20.0)	90 (17.4)	75 (24.3)	
75–84	230 (27.8)	107 (20.7)	123 (39.8)	
≥ 85	73 (8.8)	55 (10.6)	18 (5.8)	
Sex (female)	544 (65.9)	301 (58.2)	243 (78.9)	< 0.01
Relationship with patient				
Spouse	336 (40.7)	134 (25.9)	202 (65.4)	< 0.01
Biological child	392 (47.5)	305 (59.0)	87 (28.2)	
Son‐in‐law or daughter‐in‐law	84 (10.2)	69 (13.3)	15 (4.9)	
Other	14 (1.7)	9 (1.7)	5 (1.6)	

*Note:* Continuous variables are presented as medians (IQRs) and were analyzed using the Mann–Whitney *U* test, owing to their non‐normal distributions. Categorical variables are presented as n (%) and were analyzed using chi‐squared tests for comparisons between sexes. Normality was assessed using the Shapiro–Wilk test, and appropriate non‐parametric methods were applied.

Abbreviations: IQR, interquartile range; SD, standard deviation.

The level of care required by the patients was assessed according to the Japanese long‐term care (LTC) system, which categorizes care needs into seven levels: “Support Required” (Levels 1 and 2) and “Long‐Term Care Required” (Levels 1–5), with Level 1 being the least disabled and Level 5 the most [31]. Eligibility was determined using a standardized 74‐item questionnaire focused on activities of daily living (ADLs). This initial assessment was followed by a decision made by a long‐term care approval board, which included healthcare and social welfare experts. The board's decision was based on computer‐generated results, a home‐visit report, and a medical doctor's opinion. In this study, the carers reported the LTC level assigned to their patients through the questionnaire. Based on prior research, we grouped “‘Support Required” and “Long‐Term Care Level 1” into one category and “Long‐Term Care Level 2 or Higher” into another, with the latter indicating severe functional disability [32].

#### 2.3.4 Carer‐Related Information

Carer information such as sex, age, and relationship to the patient was collected through the questionnaire.

### 2.4 Statistical Analysis

Statistical analysis commenced with descriptive statistics for continuous variables (expressed as means and standard deviations) and categorical variables (expressed as frequencies and percentages). Continuous variables were analyzed using Student's *t*‐tests if they had normal distributions, and Mann–Whitney *U* tests for any with non‐normal distributions—based on the Shapiro–Wilk test for assessing data distribution normality. Specifically, the patients' ages, carers' ages, and follow‐up periods from initial diagnosis to survey were analyzed using the Mann–Whitney *U* test, because these variables did not follow normal distributions. Categorical variables were analyzed using chi‐squared tests for comparisons between sexes. Given that the Japanese MHLW provides different cancer screening recommendations for males and females, we separated the data by sex to ensure that our findings accurately reflected these differences. Cancer screening attendance rates across different age groups (65–74, 75–84, and ≥ 85 years) were calculated and compared to national data using Chi‐squared tests.

The relationship between cancer screening attendance and carer health literacy was assessed using odds ratios, with health literacy classified into low and high groups based on CCHL median scores. We first calculated unadjusted odds ratios (ORs) to evaluate the association between health literacy and screening attendance. Binary logistic regression was then used to estimate adjusted ORs (AORs) and 95% confidence intervals (CIs) while accounting for potential confounders. Each literacy component was added to the model independently, adjusting for potential confounders such as the patient's age, required level of care, and dementia type at initial diagnosis, as well as the carers' age, sex, and relationship to the patient. The theoretical justification for these adjustments was based on the well‐established influence of these factors on healthcare access and decision‐making in patients with cognitive impairment. Age and level of care have been shown to be critical determinants of both cognitive function and the ability to engage in healthcare activities [33, 34]. Dementia type has been shown to influence the progression of cognitive decline and the associated burden on carers, which in turn can impact health‐related decision‐making [35, 36]. Similarly, the demographic characteristics of the carers, such as their age, sex, and relationship to the patient, represent key variables that affect the dynamics of caregiving and the decision‐making process regarding cancer screenings [37, 38]. We initially considered including the patient's required level of care, dementia type, and the carer's relationship to the patient as potential covariates. However, the level of care did not significantly influence carer health literacy and appeared to function more as an intermediary factor. Similarly, dementia type was not associated with carer health literacy. Therefore, both variables were excluded from the final model. By including these covariates, our analysis aimed to isolate the specific impact of carer health literacy on cancer screening attendance, ensuring that the observed associations were not confounded by these critical factors. Separate analyses were performed for each cancer screening type.

## 3 Results

### 3.1 Participant Inclusion

Of the initial 5148 patients identified, 1439 did not respond to the survey invitation (“non‐responses”) and 146 declined to participate (“non‐consent”), leaving 3563 responses. After excluding 899 responses from patients, 385 from non‐primary carers, and 43 from individuals with unspecified relationships, 2236 primary carer responses were retained. Further exclusions were made for cases where the patient was deceased (*n* = 317) or had moderate‐to‐severe dementia (*n* = 1000), as well as for responses with missing key data (*n* = 70). This resulted in a final analytical cohort of 826 primary carers (Figure 2).

### 3.2 Baseline Characteristics

Among the 826 patients analyzed, 517 and 309 were women (62.6%, average age = 83.9 years) and men (37.4%, average age = 81.5 years), respectively. The median age of the primary carers was 67.0 years, and 65.9% were women. The most common relationship to their patient was biological child (47.5%), followed by spouse (40.7%; Table 1). Biological children were significantly more common among female patients (59.0%), while spouses were more prevalent among male ones (65.4%). The most frequent care requirement categories were support required at Levels 1–2 and Care Level 1 (48.1%). The most common initial diagnosis was MCI (35.7%), followed by Alzheimer's disease (34.6%). Health literacy variables were assessed using the CCHL scale. The median score for communicative health literacy was 11 (interquartile range [IQR]: 9–13); and that for critical health literacy was 7 (IQR: 6–8). Based on these median scores, the carers were dichotomized into low and high groups for each literacy component. In the communicative health literacy domain, 44.6% of the carers fell into the low group, while 55.4% were in the high one. For critical health literacy, 47.6% were in the low group and 52.4% were in the high one.

### 3.3 Cancer Screening Attendance and Its Association With Carer Health Literacy

**FIGURE 3:**
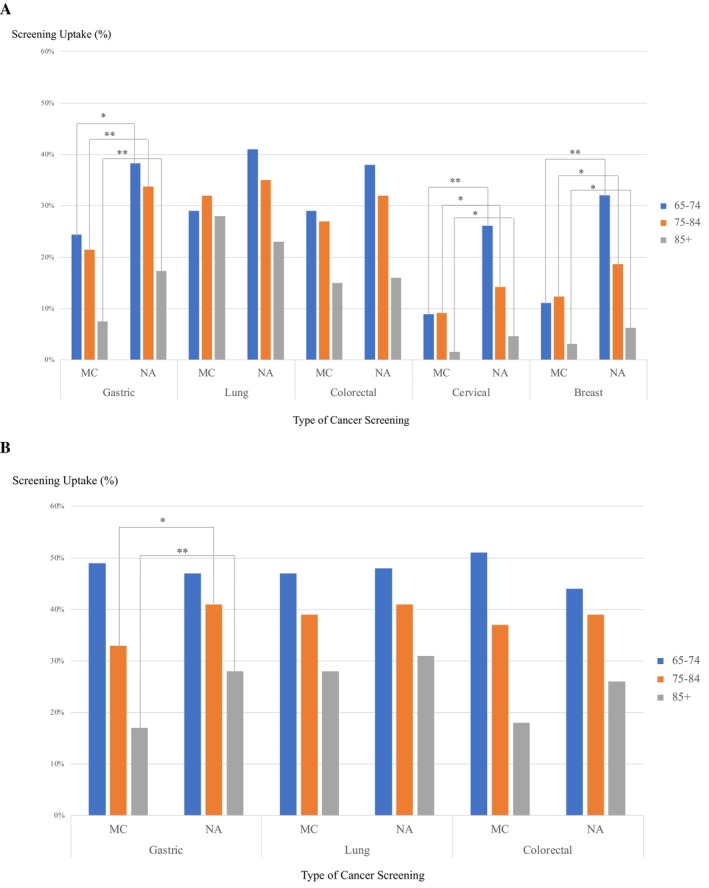
Cancer screening uptake among individuals with declining cognitive function, compared to national data. (A) Females. (B) Males. *p < 0.05, **p < 0.01, MC: memory clinic cohort, NA: national average.

The cancer screening attendance rates varied according to age and sex (Figure 3A,B). For female patients, the rates decreased with age across all recommended screenings. Gastric cancer screenings were attended by 24% of women aged 65–74 years, which dropped to 8% in those aged > 85 years. Lung and colorectal cancer screenings exhibited similar age‐related declines. Cervical and breast cancer screening attendance in the memory clinic cohort was substantially lower than the national averages—particularly for women aged 65–74 years, of whom only 11% were screened for breast cancer (a significant drop from the 32% national average). Although this disparity decreased in the oldest age groups, it remained significant. The male patients exhibited higher gastric, lung, and colorectal cancer screening rates than the female ones, particularly those aged 65–74 years. Compared to the national data from the Comprehensive Survey of Living Conditions, significantly lower attendance rates were observed in the memory clinic cohort among women across all age groups, particularly for gastric, cervical, and breast cancers. For men, significant differences were noted in the older age groups for gastric cancer screening. The most commonly reported reasons for not attending cancer screenings were “Can go to the doctor anytime if needed” (46.8%), “Was going to/was admitted at a medical facility at the time” (16.4%), “Concerned about COVID‐19 infection” (10.9%), and “No time” (8.9%).

Our analysis of the association between the health literacy levels of carers (subdivided into communicative and critical) and cancer screening attendance rates of their patients revealed higher attendance rates in the high group vs. the low one across both subscales (Tables 2 and 3). For female patients, those whose carers had higher levels of communicative health literacy were significantly more likely to attend screenings for gastric (AOR, 1.77; 95% CI, 1.03–3.04), colorectal (AOR, 1.70; 95% CI, 1.08–2.70), and breast (AOR, 3.08; 95% CI, 1.40–6.76) cancers. In male patients, a higher likelihood of attending lung cancer screenings was seen in the high health literacy group vs. the low one (AOR, 1.82; 95% CI, 1.11–2.99).

**TABLE 2:** Association between cancer screening uptake and carer health literacy (women).

		Had checkup		
	HL group (*n*)	*n* (%)	OR	AOR
Gastric cancer screening				
Communicative HL	Low (213)	26 (12.2)	—	—
	High (288)	51 (17.7)	1.55 (0.93–2.58)	1.77 (1.03–3.04)
Critical HL	Low (239)	30 (12.6)	—	—
	High (262)	47 (17.9)	1.52 (0.93–2.50)	1.60 (0.95–2.69)
Lung cancer screening				
Communicative HL	Low (208)	62 (29.8)	—	—
	High (286)	93 (32.5)	1.14 (0.77–1.67)	1.15 (0.78–1.71)
Critical HL	Low (234)	69 (29.5)	—	—
	High (260)	86 (33.1)	1.18 (0.81–1.73)	1.18 (0.80–1.73)
Colorectal cancer screening				
Communicative HL	Low (210)	36 (17.1)	—	—
	High (286)	73 (25.5)	1.66 (1.06–2.59)	1.70 (1.08–2.70)
Critical HL	Low (235)	44 (18.7)	—	—
	High (261)	65 (24.9)	1.44 (0.94–2.22)	1.39 (0.90–2.17)
Cervical cancer screening				
Communicative HL	Low (208)	10 (4.9)	—	—
	High (284)	18 (6.3)	1.34 (0.61–2.97)	1.36 (0.60–3.07)
Critical HL	Low (235)	12 (5.1)	—	—
	High (257)	16 (6.2)	1.23 (0.57–2.67)	1.20 (0.55–2.64)
Breast cancer screening				
Communicative HL	Low (211)	9 (4.3)	—	—
	High (286)	31 (10.8)	2.73 (1.27–5.86)	3.08 (1.40–6.76)
Critical HL	Low (238)	14 (5.9)	—	—
	High (259)	26 (10.0)	1.79 (0.91–3.51)	1.83 (0.92–3.68)

*Notes:* Percentages represent the proportion of participants within each health literacy group (high vs. low) who attended screenings. In our binary logistic regression models, communicative and critical health literacy were assessed independently, adjusting for patients’ age and carer demographics.

Abbreviations: AOR, adjusted odds ratio; HL, health literacy; health checkups, general health examinations/screenings; OR, odds ratio.

**TABLE 3:** Association between cancer screening uptake and carer health literacy (men).

		Had checkup		
	HL group (*n*)	*n* (%)	OR	AOR
Gastric cancer screening				
Communicative HL	Low (142)	44 (31.9)	—	—
	High (149)	48 (32.2)	1.06 (0.65–1.74)	1.15 (0.69–1.92)
Critical HL	Low (142)	41 (28.9)	—	—
	High (149)	51 (34.2)	1.28 (0.78–2.11)	1.36 (0.81–2.27)
Lung cancer screening				
Communicative HL	Low (133)	44 (33.1)	—	—
	High (151)	69 (45.7)	1.70 (1.05–2.76)	1.82 (1.11–2.99)
Critical HL	Low (137)	48 (35.0)	—	—
	High (147)	65 (44.2)	1.47 (0.91–2.37)	1.50 (0.92–2.45)
Colorectal cancer screening				
Communicative HL	Low (139)	48 (34.5)	—	—
	High (147)	53 (36.1)	1.07 (0.66–1.74)	1.20 (0.72–2.00)
Critical HL	Low (137)	48 (35.0)	—	—
	High (149)	53 (35.6)	1.02 (0.63–1.66)	1.08 (0.65–1.80)

*Note:* Percentages represent the proportion of participants within each health literacy group (high vs. low) who attended screenings. In our binary logistic regression models, communicative and critical health literacy were assessed independently, adjusting for patients’ age and carer demographics.

Abbreviations: AOR, adjusted odds ratio; HL, health literacy; health checkups, general health examinations/screenings; OR: odds ratio.

## 4 Discussion

This study compared cancer screening rates among Japanese individuals with cognitive impairment, by aligning data from a memory clinic cohort with national statistics. Significant differences were observed between the memory clinic cohort and the national average—particularly among women, who exhibited lower screening rates for gastric, cervical, and breast cancers across all age groups. Men aged > 75 years demonstrated a similar pattern for gastric cancer screenings. While individuals with dementia are generally less likely to undergo cancer screenings [39, 40], our findings indicate age‐ and sex‐specific trends, highlighting the complex factors affecting screening decisions for those with MCI and early‐stage dementia. These factors included logical challenges, inability to comprehend the importance of screenings, and varying progression of cognitive decline. This complexity necessitates targeted information and enhanced support within medical facilities to promote screening behaviors—particularly for less invasive screenings, such as lung and colorectal cancer, which may present fewer logistical challenges. Given these disparities, we recommend implementing targeted interventions to improve cancer screening rates among patients with cognitive impairment, with a particular focus on tailored communication strategies that consider both the cognitive abilities of patients and the health literacy levels of their carers.

In this study, the variation in cancer screening attendance by type may be attributable to the fact that more complex procedures, such as those conducted during gastric and breast cancer screenings [41, 42, 43], impose a greater burden on individuals with cognitive impairment and their carers [14, 15], potentially leading to reduced participation rates. For example, while mammograms are often perceived as complex, owing to the discomfort and anxiety associated with the procedure, the colorectal cancer screening method analyzed in this study refers to fecal occult blood tests, which are less invasive than procedures such as colonoscopies. This distinction is significant, as it highlights the fact that the screening methods analyzed in this study may vary in complexity and burden. Addressing issues of procedure complexity and carer burden is pivotal to improving participation rates in cancer screenings. Enhanced support within medical settings to facilitate attendance at screenings and strong advocacy for early detection are also essential. Emphasizing the relative ease of certain screenings may also encourage carers and patients alike.

In this study, health literacy in carers—particularly communicative health literacy—significantly influenced the cancer screening behaviors of patients with cognitive impairment. Considering broader trends, socioeconomic factors, and resource availability are critical to shaping these behaviors—particularly in Japan, where the rates are substantially lower than in countries with more robust cancer screening cultures [44, 45, 46]. Consistent with the collateral care concept discussed by Yoshida et al., familial factors such as financial instability and loneliness can also exacerbate barriers to seeking medical care [47]. Their research emphasizes considering the broader familial and social contexts when addressing the health needs of patients with cognitive decline [47]. In the present study, high health literacy levels among carers correlated with increased patient screening attendance and enhanced recognition of screening importance, indicating that health literacy is a critical factor influencing screening behaviors [40]. Our study emphasizes carer involvement in promoting attendance, suggesting that decision‐making support structures in healthcare settings should cater to the needs of those with cognitive decline and their carers to achieve more effective cancer screening decisions. In the context of carer health literacy and its relationship with cancer screening behaviors, we observed that communicative health literacy played a significant role, particularly in terms of promoting breast cancer screening, for which the AOR, was notably high. This finding suggests that female spouses, who are often primary carers, should understand the necessity and procedures involved in breast cancer screening. The communicative aspect of health literacy is particularly relevant, and enhancing the communication skills of carers, particularly spouses, can lead to more informed decisions and higher screening rates.

The absence of established cancer screening guidelines for individuals with cognitive decline highlights the importance of this study in the context of public health policies. With Japan's cancer screening rates lagging behind those of countries such as the US and UK by 30%–40%, the need for intervention has become crucial. The lower rates in Japan have also been attributed to systemic differences in health insurance, methods used for encouraging attendance, and potentially to societal stigma that may undervalue the health needs of individuals with dementia. In our study, common reasons for not screening included lack of time, confidence in personal health, and the belief that medical consultation is available when needed—suggesting a gap in health literacy regarding the importance of cancer screening. Our findings support the need for tailored educational campaigns and screening initiatives for individuals with cognitive impairment and their carers, to enhance both patient and family health literacy. Furthermore, as Fowler et al. suggested, developing decision‐making support frameworks for healthcare services is crucial [1]. Moreover, our study advocates for patient‐centered communication strategies, emphasizing the roles of healthcare systems in facilitating easier access to cancer screenings. Challenging stigma and improving awareness concerning the capabilities and needs of individuals with cognitive decline is essential to fostering equitable care. Based on our findings, scheduling cancer screenings in conjunction with routine care visits may help reduce barriers for patients with cognitive impairment, potentially making information more accessible and comprehensible while also minimizing logistical obstacles to attendance. This approach may merit further exploration in future studies. Through these recommendations, we aim to enhance dialog among carers, patients, and healthcare providers. Additionally, given the particular challenges related to health literacy in Japan, it is important to address misconceptions and enhance the understanding of cancer screening significance. Future policies should also consider screening approaches for individuals with disabilities, including those with dementia, to ensure equitable healthcare access and informed decision‐making.

### 4.1 Study Limitations

This study included data from a single memory clinic in Aichi Prefecture, Japan, with participants from both urban and rural areas. While this focus provides valuable longitudinal insights, the use of national data rather than a matched control group introduces potential hidden biases and limits the ability to assess health literacy at a baseline level. Furthermore, the study did not account for motivational factors or other reasons that might influence a patient's decision not to undergo cancer screenings. These elements may provide a more nuanced understanding of screening behaviors if explored in future research. Additionally, although the national data used does not include regional specifics, limiting the examination of geographical disparities, our findings still offer a critical perspective on cancer screening behaviors among patients with cognitive impairment in Japan.

### 4.2 Clinical Implications

This study revealed significant clinical implications for enhancing cancer screening participation among individuals with cognitive decline. First, targeted strategies that improve health literacy in carers are required, as this directly influences screening behaviors. Moreover, our study showed that logistical challenges significantly impact participation, particularly in screenings requiring complex preparatory steps. Streamlined processes and carer assistance—such as scheduling screenings during regular care visits, providing transportation, and simplifying preparatory instructions—may mitigate these barriers. Healthcare policies should address the specific needs of patients with cognitive decline, including developing clear guidelines for cancer screening, interventions focused on carer support, better communication strategies, and logistical simplification. Lastly, our data underscore the necessity of nuanced approaches that consider the severity of each patient's cognitive decline. For those with mild impairment, regular screening benefits outweigh the risks, given their extended life expectancy. A comprehensive healthcare framework supporting patient‐centric, informed decision‐making might bridge the screening gap for this vulnerable population.

## 5 Conclusions

While our findings suggest a gap in cancer screening rates between individuals with cognitive impairment and the general population, the effect sizes for the association between health literacy and screening rates were small and often statistically insignificant. This indicates that further research is warranted to better understand the specific barriers and develop tailored cancer screening strategies that effectively address both individual and systemic factors for this population.

## Author Contributions

**Yujiro Kuroda:** conceptualization (lead), data curation (equal), formal analysis (lead), funding acquisition (lead), investigation (lead), methodology (lead), project administration (lead), software (lead), writing – original draft (lead), writing – review and editing (lead). **Aya Goto:** investigation (equal), methodology (equal), supervision (lead), writing – original draft (supporting), writing – review and editing (equal). **Kazuaki Uchida:** investigation (equal), project administration (equal), writing – review and editing (equal). **Taiki Sugimoto:** data curation (equal), investigation (equal), writing – review and editing (equal). **Kosuke Fujita:** investigation (equal), project administration (equal), writing – review and editing (equal). **Yoko Yokoyama:** investigation (equal), project administration (equal), writing – review and editing (equal). **Takeshi Nakagawa:** investigation (equal), project administration (equal), writing – review and editing (equal). **Tami Saito:** conceptualization (equal), data curation (equal), funding acquisition (equal), investigation (equal), project administration (equal), supervision (equal), writing – review and editing (equal). **Taiji Noguchi:** data curation (equal), investigation (equal), writing – review and editing (equal). **Ayane Komatsu:** data curation (equal), investigation (equal), writing – review and editing (equal). **Hidenori Arai:** funding acquisition (equal), project administration (equal), supervision (equal), writing – review and editing (equal). **Takashi Sakurai:** conceptualization (equal), data curation (equal), funding acquisition (equal), investigation (equal), project administration (equal), supervision (equal), writing – review and editing (equal).

## Ethics Statement

This study adhered to the ethical standards required for research involving human subjects. Approval was obtained from the institutional review board of the National Center for Geriatrics and Gerontology prior to data collection. All of the participants, their legal guardians, or primary carers (in cases of participants with cognitive decline) provided informed consent to participate in this study. Data confidentiality and participant anonymity were ensured throughout the research process.

## Conflicts of Interest

The authors declare no conflicts of interest.

## Data Availability

Anonymized data supporting the findings of this study are housed within the NCGG. These data may be made available upon reasonable request to the corresponding author, contingent on approval from the NCGG's institutional review board and in compliance with institutional ethical guidelines.
